# Self-Association of an Activating Natural Killer Cell Receptor, KIR2DS1

**DOI:** 10.1371/journal.pone.0023052

**Published:** 2011-08-30

**Authors:** Michael Hayley, Sarah Bourbigot, Valerie Booth

**Affiliations:** 1 Department of Biochemistry, Memorial University of Newfoundland, St. John's, Newfoundland, Canada; 2 Department of Physics and Physical Oceanography, Memorial University of Newfoundland, St. John's, Newfoundland, Canada; Centre de Recherche Public de la Santé (CRP-Santé), Luxembourg

## Abstract

As a major component of the innate immune system, natural killer cells are responsible for activating the cytolytic killing of certain pathogen-infected or tumor cells. The self-recognition of natural killer cells is achieved in part by the killer cell immunoglobulin-like receptors (KIRs) protein family. In the current study, using a suite of biophysical methods, we investigate the self-association of an activating KIR, KIR2DS1. This KIR is of particular interest because when in the presence of the HLA-Cw6 protein, KIR2DS1 becomes a major risk factor for psoriasis, an autoimmune chronic skin disease. Using circular dichroism spectroscopy, dynamic light scattering, and atomic force microscopy, we reveal that KIR2DS1 self-associates in a well-defined fashion. Our novel results on an activating KIR allow us to suggest a working model for the KIR2DS1- HLA class I molecular mechanism.

## Introduction

The innate immune system provides immediate defense against infection. Natural Killer (NK) cells are a major component of the innate immune system and are responsible for activating the cytolytic killing of certain pathogen-infected or tumor cells. It is essential for the NK cells to discriminate between self- and non-self to ensure that NK-mediated lysis occurs against the appropriately targeted cells. The self-recognition of NK cells is achieved in part by receptors that recognize MHC class I molecules (HLA-A, HLA-B, and HLA-C) on self-cells [1]. To date, three distinct families of MHC class I – recognizing NK cell receptors have been indentified; Ly49, CD94/NKG2, and killer cell immunoglobulin-like receptors (KIRs) [2].

Killer cell immunoglobulin-like receptors are type I transmembrane glycoproteins that contain two or three extracellular immunoglobulin (Ig) domains with either long (2DL, 3DL) or short (2DS, 3DS) cytoplasmic tails [3], [4], [5]. Receptors with long tails are inhibitory, while receptors with short tails are activating. Long tail receptors contain an immunoreceptor tyrosine-based inhibitory motif (ITIM) that contributes to the inhibitory signal, and is essential for recognizing self-cells. The activating KIRs (2DS1, 2DS2, and 3DS1) are associated with DAP12, which participates in signaling events, such as cytolysis. However, much less is known about the biological role of the activating, short-tail receptors compared to their inhibitory counterparts.

Combinations of certain KIRs and MHC class I molecules, such as HLA-Cw6 and KIR2DS1, have been linked with susceptibility to autoimmune diseases, inlcuding psoriasis. Psoriasis is a lifelong skin disease that affects an estimated 1 to 3% of the world's population. Recognized as the most prevalent autoimmune disease, psoriasis is caused by inappropriate activation of the cellular immune system [6]. This disease is complex and results from an interplay between multiple genetic and environmental factors [7]. These factors lead to an aggravated state of innate immunity characterized by the activity of natural killer (NK) T cells, dendritic cells, neutrophils, and keratinocytes [8]. More specifically, the presence of the HLA-Cw6 protein in combination with its receptor, KIR2DS1, is a major risk factor for psoriasis [9]. In fact, ∼50% of psoriasis patients carry the HLA-Cw6 gene and these individuals usually have a younger age of onset and an increased disease severity [10]. Therefore, it is important to understand the molecular mechanism of KIR2DS1.

In the present work we have characterized KIR2DS1 using a suite of biophysical methods. We provide evidence that the purified extracellular portion of the protein self-associates under many different conditions. Using circular dichroism spectroscopy, dynamic light scattering and atomic force microscopy, we revealed the first direct evidence that KIR2DS1 self-associates in a well-defined fashion.

## Materials and Methods

### KIR2DS1 expression and purification

cDNA (bacteria codon optimized) encoding the extracellular region of KIR2DS1 (MAHEGVHRKPSLLAHPGRLVKSEETVILQCWSDVMFEHFLLHREGMFNDTLRLIGEHHDGVSKANFSISRMRQDLAGTYRCYGSVTHSPYQLSAPSDPLDIVIIGLYEKPSLSAQPGPTVLAGENVTLSCSSRSSYDMYHLSREGEAHERRLPAGTKVNGTFQANFPLGPATHGGTYRCFGSFRDSPYEWSKSSDPLLVSVTLVPRGS), plus a C-terminal 6-His tag, inserted into a PET23d(+) vector was purchased from GenScript (Piscataway, NJ, USA). The plasmid was then transformed into BL21 competent *Escherichia coli* cells for large scale expression of KIR2DS1. Cells were grown at 37°C to an OD of 0.6, induced with 1 mM IPTG and harvested after 3 hours.

The harvested BL21 cells were lysed using a French pressure cell and solubilized in 8 M urea, 100 mM Tris-HCl (pH 8.0) and 1 mM DTT. The resulting supernatant, containing KIR2DS1, was loaded onto an equilibrated Ni^2+^-nitrilotriacetic acid- agarose column (5 ml) and washed with five column volumes of 10 mM Tris-HCl (pH 8.0), 100 mM sodium chloride and 8 M urea. KIR2DS1 was eluted using an imidazole gradient and the purified protein-containing fractions were pooled.

The purified protein was put through a series of dialysis steps, following the method of Stewart *et al.* [11], to ensure proper refolding. The denatured protein in 8 M urea was intially dialyzed against a series of 4 buffers containing 20 mM Tris-HCl (pH 7.8), 150 mM sodium chloride, 400 mM arginine, 1 mM β-mercaptoethanol and decreasing urea concentrations of 4, 3, 2 and 1 M. The protein was then dialyzed against 0.5 and 0 M urea in 20 mM Tris-HCl (pH 7.8), 150 mM sodium chloride, 400 mM arginine, 1 mM β-mercaptoethanol, 5 mM reduced glutathione, 0.5 mM oxidized glutathione and 0.4 mM sodium azide. The last step was dialysis against 10 mM Tris-HCl, 150 mM sodium chloride and 0.4 mM sodium azide.

### Microdrop Sceeening

Microdrop screening was used to detect KIR2DS1 aggregation as described by Lepre and Moore [12]. We tested 23 different buffer conditions with a 24 well tissue culture plate and siliconized glass cover slips. 1 mL of each buffer was pipetted into each reservoir, and then, 2 µL aliquots of protein solution in starting buffer were pipetted onto the glass coverslips. 1 µL of each of reservoir buffer was added to each drop, and the solutions were mixed by gently drawing and expelling the solution in the pipette tip. The glass slips were then inverted and sealed onto the wells using petroleum jelly. The plate was allowed to rest undisturbed at room temperature so that vapor diffusion could take place. Precipitate in the drops were detected by placing the tray against a black background, iluminating it from the slide, and visually examining each drop under a microscope. Under these lighting conditions, precipitate appears as a white spot against the black background.

### Size Measurements

The size of KIR2DS1 was measured in a Zetasizer Nano ZS instrument (Malvern Instruments Ltd., Worcestershire, U.K.) in the presence of 10 mM Tris-HCl, 150 mM sodium chloride and 0.4 mM sodium azide. Measurements were taken at 25°C.

### Circular dichroism spectroscopy

Circular dichroism spectra in the far-ultraviolet were recorded using a Jasco-810 spectrapolarimeter (Applied Photophysics, UK). The absorbance at 222 nm of the protein/reagents mixture was checked to ensure that it did not exceed an optical density of 1.0. A 1 mm cell was used and spectra were collected between 200 and 250 nm. Baselines were established using the appropriate buffer (10 mM Tris-HCl, 150 mM sodium chloride and 0.4 mM sodium azide) and 5 spectra were collected for each sample and averaged. Secondary structure content was calculated from the collected spectra using a computer program following the method of Hennessey and Johnson [13].

### Atomic force microscopy (AFM)

All experiments were conducted in a solution of 10 mM Tris-HCl, 150 mM sodium chloride and 0.4 mM sodium azide on freshly cleaved mica surfaces at room temperature. Imaging was performed on an MFP-3D (Asylum Research Inc) in contact mode using gold-coated, silicon cantilevers (Micromasch). Images are presented with minimal post-processing with a simple planefit used to correct for curvature.

## Results

### KIR2DS1 conformation

**Figure 1:**
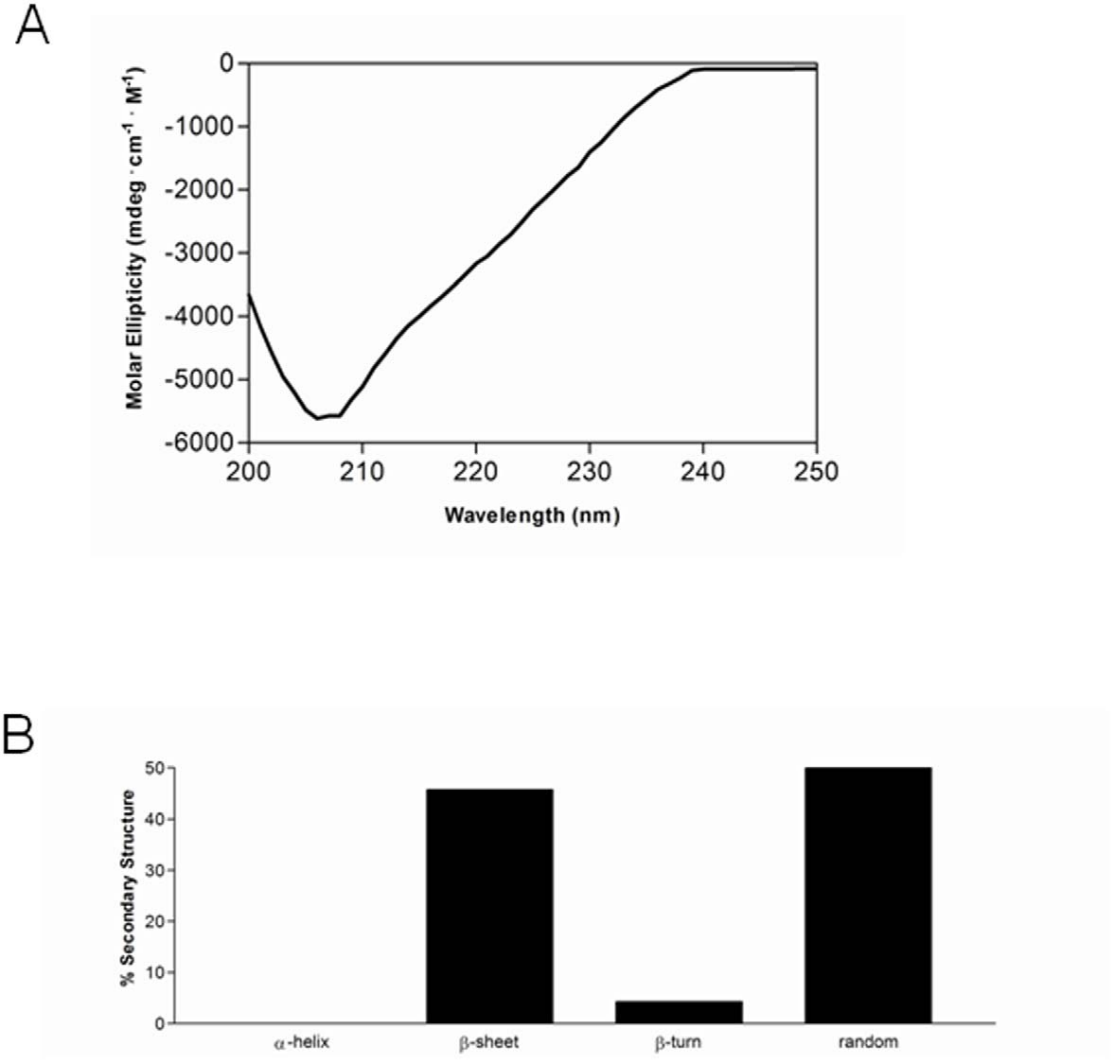
(A) Far-UV circular dichroism spectra of KIR2DS1 (3 µM, freshly refolded). (B) Secondary structural content of KIR2DS1. Secondary structural content was calculated as described in Materials and Methods.

A freshly refolded KIR2DS1 sample was centrifuged and the supernatant (3 µM) was retained. A circular dichroism spectrum of the above sample was recorded at pH 7.5 and 25°C (Figure 1A). Secondary structure content was calculated from the CD-spectrum using a computer program that followed the method of Hennessey and Johnson [13] (Figure 1B). As expected, the secondary content was composed entirely of β-structure and random coil and was devoid of α-helical structure.

### KIR2DS1 Oligomerization

During the refolding (see Materials and Methods) of KIR2DS1, visible protein aggregates was always observed in the final dialysis step containing 10 mM Tris-HCl, 150 mM sodium chloride and 0.4 mM sodium azide. We therefore carried out an extensive search for buffer conditions that would promote greater solubility of KIR2DS1, following the method of Lepre and Moore (1998). Seven buffer species, pH, salt, and TFE concentration were all varied and in total, 23 different buffer conditions were assessed (Figure S1). However, in all cases, no differences in the level of visible aggregation were observed. Additionally, no protein peaks were observed in solution NMR spectra that were acquired for several of the samples, indicating there was no substantial portion of the sample with a molecular weight sufficiently small to observe via this technique. The insensitivity of the observed aggregation to changes in buffer conditions suggested this behaviour was perhaps not the result of the commonly encountered situation where a protein behaves non-ideally in a laboratory context, but could instead derive from a well-defined, possibly functional self-association.

**Figure 2:**
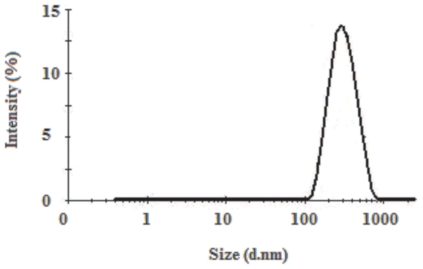
Particle size distribution of KIR2DS1.

To probe this possibility, we carried out dynamic light scattering to determine the average apparent hydrodynamic diameter of the extracellular portion of KIR2DS1 in solution, using a Zetasizer Nano ZS instrument. The size distribution calculated by the Nano software is derived from a non-negative least squares (NNLS) analysis, and it was found that KIR2DS1 (3 µM) in a 10 mM Tris-HCl, 150 mM sodium chloride and 0.4 mM sodium azide buffer had a mean hydrodynamic diameter of 320 nm (Figure 2).

**Figure 3:**
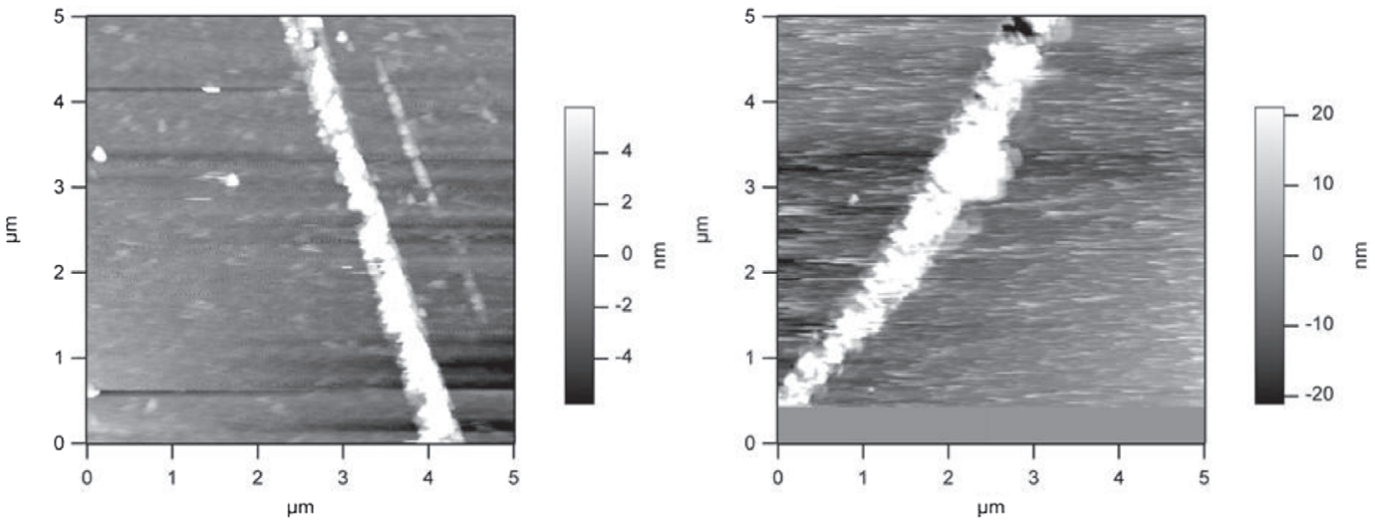
Atomic force microscopic analysis of KIR2DS1 oligomerization. Two representative images are presented.

Since the hydrodynamic diameter measured by DLS depends on the shape, as well as the size of the particles under study, we next proceeded to use atomic force microscopy to visualize the self-association of KIR2DS1 (Fig. 3). In all scanned regions, KIR2DS1 self-associated in rod-like shapes of similar dimensions, suggesting that the KIR2DS1 self-association is a well-defined process. The measured dimensions indicate that the diameter of the self-associated KIR2DS1 on the mica surface is 340 nm.

**Figure 4:**
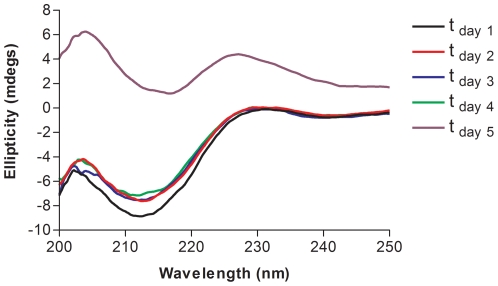
KIR2DS1 oligomerization time course study. Circular dichroism spectra were aquired over a period of 5 days on the same KIR2DS1 sample.

**Figure 5:**
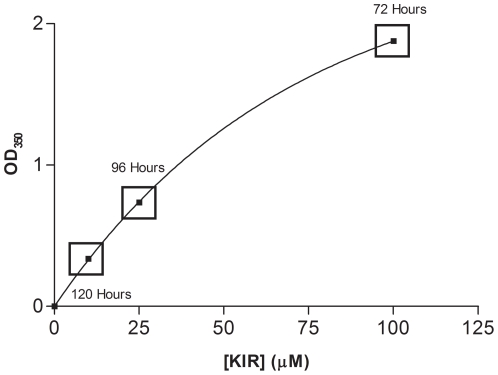
The concentration dependence of KIR2DS1 oligomerization. Optical density measurements at 350 nm were conducted on various concentrations of KIR2DS1 (10, 25 and 50 µM). The hours denote the time, after placing the protein in the first refolding buffer, that visible aggregates were observed.

As mentioned earlier, during the refolding of KIR2DS1, visible protein aggregates are observed in final dialysis step. To investigate the self-association in further detail, we carried out a time course measurement using circular dichroism spectroscopy immediately after refolding. A freshly refolded KIR2DS1 sample was centrifuged and the supernatant was concentrated to a final concentration of 5 µM in 10 mM Tris-HCl, 150 mM sodium chloride and 0.4 mM sodium azide. The protein sample was then monitored for a period of 5 days. After the first day, small changes can be observed in the spectrum (Fig. 4). For the next 3 days the secondary structure of KIR2DS1 remained constant. However, on the fifth day, the intensity of the spectrum decreased by ∼85% (Fig. 5). The decrease in the intensity of the spectrum is most likely the result of KIR2DS1 self-association, and eventually, aggregate formation of the protein. We speculate that the self-associated KIR2DS1, whose surface area is less exposed than it would be for the same concentration of free, non-associated protein, results in a reduced total amount of the optically active protein available and hence, a reduction in the intensity of the spectrum.

The KIR2DS1 self-association process is also dependent upon its concentration, as determined by light scattering at 350 nm (Fig. 5). It can be seen in Figure 5 that higher concentrations of KIR2DS1 exhibit an increased degree of light scattering, suggesting that the self-association of these samples are increased. Also, higher concentrations of KIR2DS1 showed visible signs of aggregation at earlier time points during the refolding of the protein (100 µM – 72 hours (3 M urea); 25 µM – 96 hours (2 M urea); 10 µM – 120 hours (1 M urea). Aggregation now occurs at earlier time points due to the fact that the concentration is increased.

## Discussion

To date, there has been no study that has directly examined the structure and/or self-association of KIR2DS1. Snyder *et al.* [14] have designed a model of KIR2DL2 oligomerization based on the protein-protein contacts observed in the crystal [15]. KIR2DS1 shares ∼90% homology with the above members (KIR2DL1 and KIR2DL2) and it would seem realistic that KIR2DS1 would self-associate as well.

Circular dichroism spectroscopy revealed that the KIR2DS1 is devoid of α-structure and contains a mixture of ß- and random structure consistent with the previously crystallized KIRs (KIR2DL1, KIR2DL2, KIR2DL3, KIR2DS2, KIR2DS4) [15], [14], [16], [17], [18]. In contrast to x-ray crystal structures where the protein is not studied in solution, we applied a variety of techniques to KIR2DS1 in solution. Using CD, DLS and AFM, we provide the first evidence for the self-association of KIR2DS1 in solution. More importantly, results from the AFM data reveals that KIR2DS1 self-associates in a rod-like shape and suggests that this self-association is a well-defined process.

**Figure 6:**
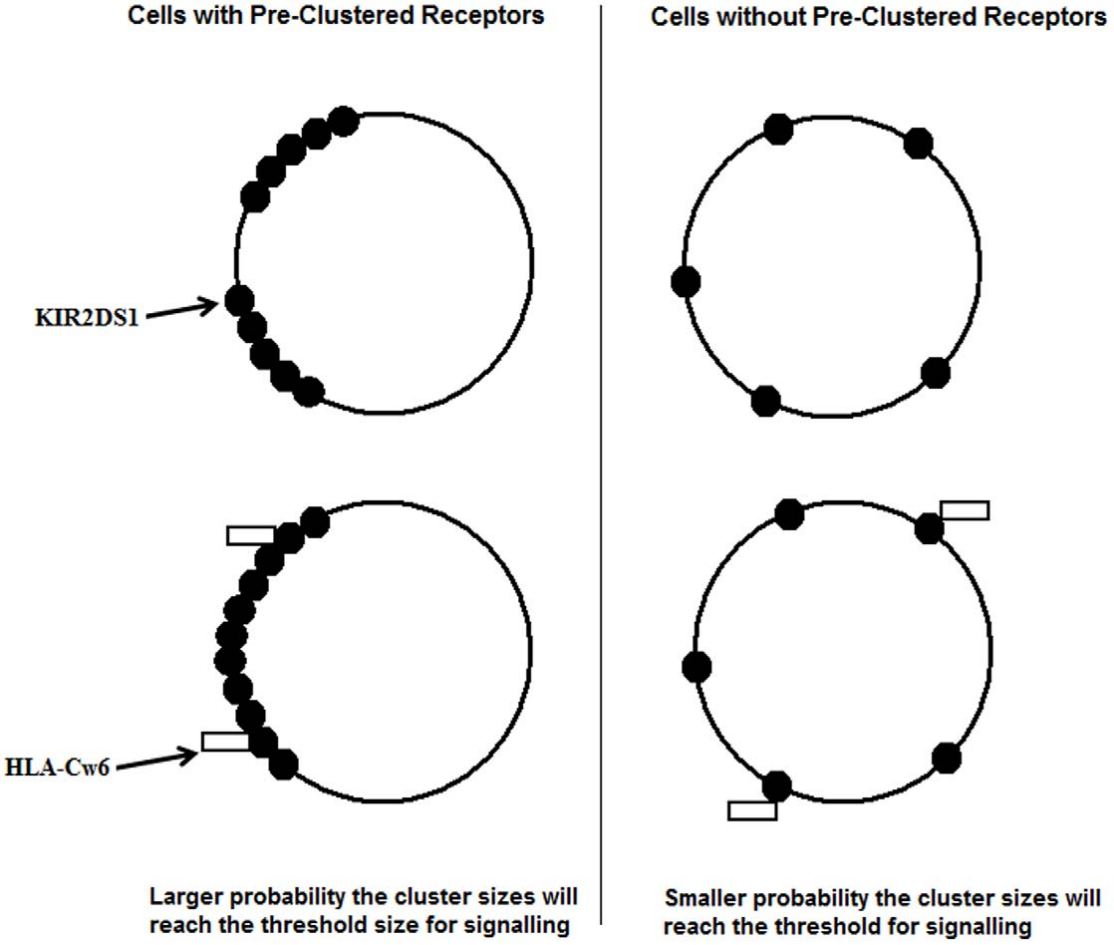
Working model for the role of KIR2DS1 in innate immunity.

Ligand-induced receptor aggregation is a common mechanism for receptor-mediated signalling in immune responses [19], [20]. However, although this may be assumed to be the case for KIRs, there is very little or no evidence that this occurs in these receptors. The results presented here allow us to define a working model for the KIR2DS1- HLA class I molecules. The innate immune system is one of the first defense strategies, and in such a case, ligand-induced receptor aggregation on the surface of NK cells would appear to be inefficient. First of all, once an antigen enters the host system, the NK cell and the antigen have to be close enough in proximity for the antigen-receptor interaction to occur. Secondly, the NK KIR2DS1 receptors would have to be localized to a common region in order to initiate the aggregation process, which in turn, would lead to activation of DAP12 signalling cascade. Alternatively, our working model proposes that the KIR2DS1 receptor oligomerize prior to their interaction with their HLA-Class I counterparts (Fig. 6). In this model, the interaction between antigen and “self-associated” KIR2DS1 then leads to the activation of DAP12 signalling cascade.

## Supporting Information

Figure S1 **Examination of KIR2DS1 solubility under various conditions.**
(DOC)
