# Supplementary material for: Self-Association of an Activating Natural Killer Cell Receptor, KIR2DS1
Source: PLoS One. 2011 Aug 30;6(8):e23052. doi: 10.1371/journal.pone.0023052 (PMC3166062; doi:10.1371/journal.pone.0023052)
Supplement: Figure S1 — Examination of KIR2DS1 solubility under various conditions. (DOC) [file pone.0023052.s001.doc]

| **Buffer** | **pH Tested** | **NaCl (mM)** | **TFE (%)** |
| --- | --- | --- | --- |
| Tris | 7.5 | 100 | - |
| Tris | 7.5 | 150 | - |
| Tris | 7.5 | 200 | - |
| Tris | 7.5 | 300 | - |
| Tris | 7.5 | 150 | 5 |
| Tris | 7.5 | 150 | 10 |
| Tris | 7.5 | 150 | 25 |
| Tris | 8.0 | 150 | - |
| Tris | 8.5 | 150 | - |
| Sodium Phosphate | 6.0 | 150 | - |
| Sodium Phosphate | 6.5 | 150 | - |
| Sodium Phosphate | 7.5 | 150 | - |
| Potassium Phosphate | 6.0 | 150 | - |
| Potassium Phosphate | 6.5 | 150 | - |
| Potassium Phosphate | 7.0 | 150 | - |
| Mes | 5.5 | 150 | - |
| Mes | 6.0 | 150 | - |
| Mes | 6.5 | 150 | - |
| HEPES | 7.0 | 150 | - |
| HEPES | 7.5 | 150 | - |
| HEPES | 8.0 | 150 | - |
| Sodium Acetate | 5.5 | 150 | - |
| PBS | 7.5 | 150 | - |
